# Preschool Children Fail Primate Prosocial Game Because of Attentional Task Demands

**DOI:** 10.1371/journal.pone.0068440

**Published:** 2013-07-03

**Authors:** Judith Maria Burkart, Katja Rueth

**Affiliations:** Anthropological Institute and Museum, University of Zurich, Zurich, Switzerland; University of Florence, Italy

## Abstract

Various nonhuman primate species have been tested with prosocial games (i.e. derivates from dictator games) in order to better understand the evolutionary origin of proactive prosociality in humans. Results of these efforts are mixed, and it is difficult to disentangle true species differences from methodological artifacts. We tested 2- to 5-year-old children with a costly and a cost-free version of a prosocial game that differ with regard to the payoff distribution and are widely used with nonhuman primates. Simultaneously, we assessed the subjects’ level of Theory of Mind understanding. Prosocial behavior was demonstrated with the prosocial game, and did not increase with more advanced Theory of Mind understanding. However, prosocial behavior could only be detected with the costly version of the game, whereas the children failed the cost-free version that is most commonly used with nonhuman primates. A detailed comparison of the children’s behavior in the two versions of the game indicates that the failure was due to higher attentional demands of the cost-free version, rather than to a lack of prosociality per se. Our results thus show (i) that subtle differences in prosociality tasks can substantially bias the outcome and thus prevent meaningful species comparisons, and (ii) that like in nonhuman primates, prosocial behavior in human children does not require advanced Theory of Mind understanding in the present context. However, both developmental and comparative psychology accumulate increasing evidence for the multidimensionality of prosocial behaviors, suggesting that different forms of prosociality are also regulated differentially. For future efforts to understand the evolutionary origin of prosociality it is thus crucial to take this heterogeneity into account.

## Introduction

Humans stand out among primates with regard to their prosocial behavior, and recent years have seen major efforts to better understand the evolutionary origin of proactive prosociality through comparative assessment of that trait across species. Proactive prosociality (i.e., the economists’ other-regarding preferences [1]) refers to intrinsically motivated prosocial behaviors that occur spontaneously and are not solicited by the recipients through direct requests or signaling of need. It has been documented in several primate species, based on experiments derived from the economic games typically played with human subjects. Interestingly, the primate species that are most closely related to humans are not the ones which score highest in such games (reviewed in [2], [3], [4]) which suggests that phylogenetic proximity to humans is not an important explanatory variable for variation in primate proactive prosociality. Rather, convergent selection pressures must be at work, such as the impact of extensive allomaternal care [5].

However, valid inferences about the evolutionary origin of any trait, including proactive prosociality, that are based on the comparative approach critically require accurate measurement of the trait across species. The use of identical paradigms and procedures is an important first step in doing so. If such paradigms reveal similar prosocial responses in different species, including humans, it then becomes informative to have a close look at the psychological regulation, for instance with regard to the motivations underlying the prosocial behavior or the necessity of Theory of Mind understanding. Unfortunately, it is currently difficult to disentangle true species differences in prosociality from methodological artifacts, because different versions of the predominant paradigm to assess proactive prosociality, also referred to as prosocial games [6], have been used for different species. Here, we investigate how young human children perform in two commonly used versions of the prosocial game when tested under conditions identical to nonhuman primates (i.e. non-verbally, without instructions, same procedures), and compare the psychological regulation involved in their responses to other primate species that behave prosocially in such games.

In humans, proactive prosociality is mostly assessed with dictator games, i.e. anonymous one-shot interactions in which a player receives an amount of money and has the opportunity to share any portion of it with a recipient. Players often choose non-zero contributions for the recipient, and thus show proactive prosociality (e.g. [7]). Anonymous one-shot interactions are thought to remove the possibility that players decide to give some of the money to the recipient based on motives other than proactive prosociality. Anonymity ensures that players are not responding to solicitation by the recipient (signs or signals of need, or even harrassement) and thus show reactive prosociality. The experiments are one-shot, so players will not expect the recipient to reciprocate the favor in the future.

Nonhuman primate adaptations of the dictator game, the prosocial games, typically give subjects the choice between different payoff distributions (e.g. one piece of food for ego and one for the partner [1,1] vs. one piece for ego and none for the partner [1,0], Table 1). Importantly, the choices of the subject are then compared to its choices in a control condition when the partner is absent, to control for a simple preference for choosing a larger amount of food (i.e. [1,1]) even if in the end, they only get part of it. A prosocial effect is detected if subjects choose the prosocial option (in this case, [1,1]) more often when a partner is present than when it is absent. The distributions are typically offered physically and the subjects can chose by instrumentally pulling a tray within reach, but token-exchange versions have been successfully implemented too [8], [9], [10].

**Table 1:** Prosocial games played with nonhuman primates.

Species	Payoff-distribution	Costly?	Prosocial effect?
Chimpanzee	1,0/1,1	no	no^1^
Chimpanzee	1,(1)/1,1^a^	no	no^2^
Chimpanzee	0,(1)/0,1^a^	no	no^2^
Chimpanzee	1,0+0,1^b^	yes	no^3^
Common marmoset	0,0/0,1	yes	yes^4^
Capuchin monkey	1,**1**/1,1^c^	no	yes^5,^ d
Capuchin monkey	**1**,1/**1**,**1** c	no	yes^5,^ d
Capuchin monkey	**1**,**1**/**1**,1^c^	no	yes/no^6,^ e
Capuchin monkey	1,**1**/1,1^c^	no	yes/no^6,^ e
Cottontop tamarin	1,0/1,1	no	no^7^
Cottontop tamarin	0,1/0,0	yes	no^7^
Cottontop tamarin	1,(3)/1,3^a^	no	no^8^
Cottontop tamarin	0,(3)/0,3^a^	yes	no^8^
Long-tailed macaque	1,(1)/1,1 a	no	yes/no^9,^ f

Studies differ with regard to **payoff distribution,** and whether help is **costly**. Included are studies only in which subjects have to choose between physically presented payoff distributions by pulling an apparatus within reach.

Ref.: ^1^Silk et al. 2005, ^2^Jensen et al. 2006, ^3^Vonk et al. 2008, ^4^Burkart et al. 2007, ^5^Lakshminarayanan and Santos 2008, ^6^Takimoto et al. 2010, ^7^Cronin et al. 2009, ^8^Stevens 2010, ^9^Massen et al. 2010.

a reward written in „()“ goes to an empty compartment and is therefore out of reach for both subjects.

b donor is allowed to choose both distributions during one trial.

c **1** = favored reward; 1 = less favored reward; 1 = non-favored reward.

d tested one-tailed t-test; but not statistically significant if tested two-tailed like other studies did.

e yes for subdominant recipient, no for dominant recipient; no for subdominant if invisible, neg. for dominant if invisible.

f yes for kin partner; no for non-kin partner; under both conditions prosocial tendency declined with increasing rank number.

Since it is not possible to test nonhuman primates in anonymous one-shot interactions, further analyses have been added to distinguish between proactive and reactive prosocial responses. To qualify as proactive, prosocial responses must not be prompted by recipients and thus also (even though not exclusively) occur in the absence of any solicitation and signaling of need, such as begging or reaching attempts [11]. The possibility for reciprocation is removed by testing dyads in only one direction and by observing the participants’ behavior immediately after the experiment. In both human and nonhuman studies, it remains difficult to fully exclude this possibility since in humans, the subject may have a hardwired disposition to always expect repeated interactions [12] or in nonhuman primates, reciprocation may go unnoticed because it occurs after post-experimental observations. However, at least the latter scenario is rather unlikely in nonhumans because delayed reciprocation is supposed to be cognitively demanding [13], [14]. Indeed, it has been shown that in tamarins, prosocial behavior emerges independently of reciprocity [15], and explicitly offering the possibility to reciprocate does usually not lead to systematically more prosocial choices in chimpanzees [16], [17], [18] and also not in children younger than 4.5 years [6].

The first aim of this study was to assess the validity of different versions of prosocial games by presenting them to young human children at an age when a broad range of prosocial behaviors, including proactive prosociality, is already established (reviewed in [19], [20], [21]). Nonhuman primates have been tested with cost-free and costly versions of the game, most commonly instantiated with the payoff distributions [1,0/1,1] or [0,0/0,1], or variations of them that include more vs less preferred foods (Table 1). Crucially, in [1,0/1,1] or cost-free settings, the donor always receives a reward but can opt, as a no-cost side effect, to also provide a reward to the recipient. In contrast, in [0,0/0,1] or costly versions, the donor never receives anything for itself, but can provide food at some small cost, e.g. by pulling a tray representing the [0,1] reward distribution within the recipient’s reach. It has been argued that motivationally, the costly choice should be more demanding, as it requires a higher degree of prosociality. Cognitively, however, the cost-free option should be more demanding because the subject has to focus attention on more than one piece of food simultaneously [11], which may potentially lead to false negative results.

If subjects pass the costly version, but fail the cost-free one, they are prosocial but are confused by some aspect of the second version (e.g. the presence of multiple pieces of food). If they pass the cost-free, but fail the costly test, they are prosocial, but only if it has no costs. Thus, if payoff distributions don’t matter, as implicitly assumed by current comparative approaches, the children should show correlated performance in both tasks, provided they have a prosocial tendency. If the children show a stronger prosocial effect in the costly version, this would reflect their young age (possibly compromising attentional demands) and the strong prosocial tendencies of children in general.

Whenever the children choose the prosocial option more often when a partner is present in either version, the psychological regulation of this behavior can also be addressed. Following the logic applied in nonhuman primate studies, assessing the role of solicitation by recipients (signs and signals of need, in human subjects including verbal requests, verbal negotiation of reciprocation) will help disentangle reactive from proactive forms of prosociality.

The second aim of our study was to assess whether the performance of human children in the prosocial game is related to their ability to understand others’ mental states, i.e. level of explicit Theory of Mind (ToM) understanding. Intuitively, an intricate link between prosociality and ToM-abilities exists: our decisions whether and how to help others often include explicit consideration of their needs, desires or beliefs. Indeed, empirical data from human children suggests that such a link may indeed exist, e.g. between mirror self-recognition and reactive comforting behavior [22], [23], [24], and between the understanding of mind and emotions and prosociality in preschoolers, with prosociality being assessed by teacher ratings [24], [25] or verbally with regard to future-oriented prosocial choices [26].

However, prosociality is far from being a unitary phenomenon: different kinds of prosocial behaviors follow different developmental trajectories without being correlated to each other [27], [28], and are also supported by different neural substrates [29]. Accordingly, the role of ToM-understanding has to be addressed for each separately. Our particular focus on proactive prosociality is based on evidence suggesting that phylogenetically, this trait may have arisen in humans non-cognitively. The overrepresentation of cooperatively breeding primates among species that show proactive prosociality both in prosocial games and also under naturalistic conditions [4] suggests that in humans too, proactive prosociality may have arisen as a side effect of cooperative offspring care, rather than resulted from highly advanced, uniquely human socio-cognitive abilities [30], [31]. If so, many of our unique socio-cognitive capacities can more parsimoniously be understood as a consequence of proactive prosociality, rather than vice versa [5], [32], [33].

Proactive prosocial behavior in nonhuman primates, assessed via social games, are highly unlikely to require explicit ToM-abilities as a prerequisite since to date, there is no evidence that nonhuman primates fully possess this ability. However, some more basic ToM-related abilities have been reported for some nonhuman primate species. Nevertheless, the distribution of prosociality measured in prosocial games among non-human primates (see Table 1), though confusing, does not support the idea that such more basic abilities linked to ToM are a limiting factor: The most reliable evidence for prosocial choices in such games come from capuchin and callitrichid monkeys (marmosets and tamarins), rather than from chimpanzees, who have more powerful ToM-abilities compared to the first two species, e.g. with regard to mirror self-recognition, which is present in chimpanzees [34] but not in cotton-top tamarins [35], marmosets [36] or capuchin monkeys [37], or with regard to visual perspective taking, which shows the same pattern of positive results in chimpanzees [38], and negative results in capuchin [39] and marmoset monkeys [40]. Thus, across nonhuman primates, there is no link between ToM-related abilities and the outcome of prosocial games.

Given these species differences, if in human children, explicit ToM-reasoning turns out to be a precondition for behaving prosocially in the prosocial games, this would suggest that we measure a trait that is only superficially similar to nonhuman primates. Furthermore, this outcome would be consistent with the idea that prosociality is the result of our derived socio-cognitive abilities [30], [31]. Alternatively, explicit ToM-reasoning may not be a precondition for and not promote proactive prosocial behavior. Together with the nonhuman primate data, this would be consistent with the idea that proactive prosociality rather enabled the emergence of uniquely human social cognition, both ontogenetically and phylogenetically [5], [32], [41].

## Methods

### Subjects

46 children (23 males, 23 females) were tested in three day-care centers located in Zurich, Switzerland. The children were between 1.5 and 5 years old (mean = 3.47, sd = 0.73). 31 dyads were composed and tested in one direction only to exclude reciprocity effects. The children knew each other and dyads were composed of same-age (median age difference in dyads: 3.7 months) and same-sex partners whenever possible. The older subject within each dyad played the donor role [42]. We included participants as donors from an age of 2 years, which resulted in a mean age of donors of 3.8 years (girls, sd = 0.54, n = 13) and 3.4 years (boys, sd = 0.57, n = 18), respectively. Thus, 70% of the donors were between 3 and 4 years old, an age range for which we expected high variation in ToM understanding [43]. Children who participated both as donor and recipient, did so first as donors, to avoid carry-over effects.

The relationship quality of the dyads was rated by nursery teachers as neutral (21 dyads), positive (9 dyads) or negative (1 dyad). The parents filled out questionnaires that allowed us to calculate the socio-economic status (SES) (assessed according to [44]) and gave information about the presence of siblings and sibling position. The experiments were approved by the Ethik-Kommission of the Kinderspital Zürich, Unterkommission SPUK. The parents gave written informed consent for the childrens’ participation.

### Prosociality Tasks

#### Setup and apparatus

**Figure 1:**
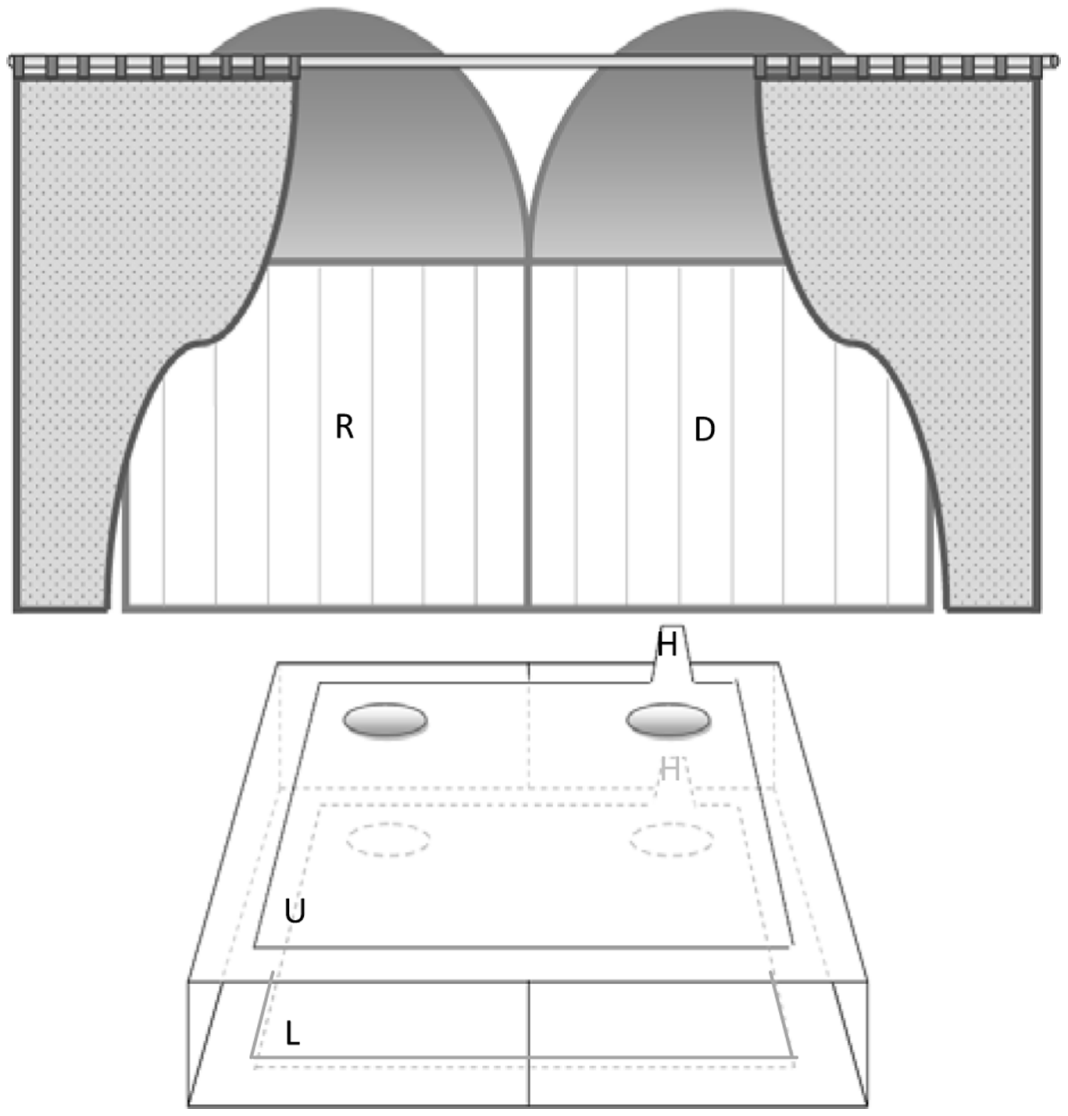
Experimental setting. Two playpens serve as compartment for the donor (D) and the recipient child (R). The handles (H) of the apparatus can be manipulated from the donor compartment only, and allow to pull the boards (U: upper board; L: lower board) with the dishes within reach. Between trials, the curtains are drawn.

The experimental setup and procedure were directly modeled after that used for marmoset monkeys [11]. Two identical playpens were used as house-like compartments, one for the donor and one for the recipient (Figure 1). The compartments were separated by an opaque divider with a window that allowed visual contact between donors and recipients. Payoff-distributions were presented on a movable apparatus with two stacked trays. On each tray two dishes were attached, one at the donor- and one at the recipient side. The donor, but not the recipient, could pull the trays within reach of both participants because handles extended from the trays into the donor compartment. In each trial, the donor could only pull one tray, because pulling one tray automatically blocked the other one. A curtain was placed in front of the playpens at a distance of circa 1.5 m and the entire apparatus could be moved forward to the playpens and backward behind the curtain. This allowed the experimenters to bait the apparatus out of view of the subjects between trials. As reward we used fruits in the training phase and sweets (Smarties) or salty snacks during the experiment, according to individual preferences of both dyad partners.

#### Procedure

A trial started with an experimenter (E) saying “ta ta ta taaa” and opening the curtain. The apparatus was moved towards to the compartments, which gave the donor the opportunity to access the handles. After the participant had pulled one handle or after a delay of 15 sec, a bell signaled the end of the trail. The apparatus was moved back behind the curtain and the curtain closed.

The experiment started with a warm-up phase, where participants had the opportunity to get used to the presence of the apparatus. They were allowed to freely explore especially the house –like compartments for 10 min and shown that they could be separated with the help of a partition. Next, we conducted a demonstration phase where the experimenter was in the donor compartment, and run a trial that was presented by a second experimenter. The demonstration consisted in the experimenter pulling a reward on its own side within reach and taking this reward. The demonstration phase was necessary because pilot trials revealed that unlike the marmoset monkeys, many children were reluctant to enter the playpens. Following the demonstration, we tested whether the child liked the reward. A plate with rewards was offered and the experimenter asked if she liked the reward and wanted a piece. The test was passed if the child took a reward, and followed by the training phase after which the children had to be able to handle the apparatus, and understand the consequences of pulling the trays. During the training phase, the partition between the compartments was removed and the subjects thus had access to both compartments. For the costly payoff distribution (see below) a reward was placed on one of the four dishes and by pulling the correct handle the children could make the reward available for themselves, either in the donor compartment, or in the recipient compartment. If participants did not pull the correct tray in each position at least twice in a row in 20 trials they were excluded from the study or were assigned to be recipients only. Once they had passed this criterion, the partition was placed between the compartments. Now the subject could experience that she could no longer reach the reward if it was placed on the recipient side of the trays and that even if pulling the tray with the reward, they would not be able to obtain it. The procedure was identical for the cost-free payoff distribution, with the exception that during the training phase, the participants had to learn to maximize their reward. Thus, three pieces of food were placed, two on either board on the donor side and one on one board on the recipient side and the participants had to pull the [1,1] distribution rather than the [1,0].

During the experiment, the compartments were separated by the partition. In the experimental condition, a partner was present in the recipient compartment, but in the control condition, the recipient compartment was empty. Each test and control condition had 9 trials. Half of the dyads were first tested with the test condition, the other half with the control condition. All training and experimental phases were videotaped. Importantly, nursery teachers were absent during testing, to minimize the possibility that the children behaved prosocially to fulfill the expectations of these authority figures.

#### Payoff distributions

Each dyad was tested with two versions of the game. In the costly version, a reward was placed in one of the four dishes, alternately on the upper or lower tray. The reward was on the recipient side during the test trials, and the subjects could choose to pull the board with the reward within reach of the recipient, pull the empty board, or not pull at all. During the first, the fifth, and the ninth trial, the reward was placed on the donor side (as motivation trials), to keep the donors engaged in the task as had previously been done for nonhuman primates. In the cost-free version, the subjects could choose between a tray baited with a reward for themselves but nothing for the recipient, or one containing both a reward for themselves and the recipient. Half of the dyads were first tested with the first distribution, the other half with the other one.

#### Data coding

We recorded the response of the donor (pulling the baited tray, the empty tray, or not pulling) during the experiments and verified the coding twice afterwards by analyzing the video tapes. Additionally, we coded the latency until the donor child had pulled, and whether or not he had looked at the partner’s plate before pulling. Furthermore, we coded the recipient’s behavioral (looking at the reward, reaching for the reward) and verbal (requests for help) signs and signals of interest in the reward before pulling by the donor. We only recorded those signs and signals that could be perceived by the donor. These signals were coded per trial as present or absent. After the recipient had taken the reward provided by the donor, we also coded reactions by donors: the donor could not attend to the recipient taking the reward, neutrally observe the recipient, or observe her taking the reward with a positive emotional reaction (smiling, verbal comments). Negative emotional reactions did not occur. 10% of all trials were coded by a second rater. Reliability of the response of the donor was complete (Cohen’s Kappa = 1). Latencies where highly correlated between the raters with 93% of the variation explained (n = 53, p<0.001), and the reliability for the behavioral coding (signaling by the recipient and reactions by the donors) was Cohne’s Kappa = 0.8.

### Theory of Mind Tasks

#### Materials and procedure

We used a test battery of four socio-cognitive tasks originally developed by Wellman and Lu [45] to assess the extent to which the children were aware of the fact that desires, beliefs or knowledge of others can differ from their own. A German version of these tests had been developed and validated by Hofer and Aschersleben [46]. The tests consist of illustrated short stories and could be ranked by increasing difficulty on a Guttman scale (diverse desire, diverse belief, knowledge access, content false belief) by Wellman and Lu [2004] and Hofer and Aschersleben [2007]. A Guttman scale assumes that if one passes a particular task, she also passes the lower ranked tasks. Four tasks were presented in a single session for each child, in a separate room at the day-care centers. Each of the tasks was coded as passed or failed.

To validate that the performance in the ToM tasks can indeed be ranked with increasing difficulty in our sample, we first used Rasch analyses to validate the order of item difficulty calculated with the Guttman Scale, as previously done by Wellman and Liu [45] and Kristen et al. [47]. The dichotomous Rasch Model is a probabilistic approach which estimates a person’s ability and item difficulty. If a person’s ability is equal to item difficulty, then the person passes this task with a probability of 0.5. If a person’s ability exceeds the item difficulty, she passes this task with a probability higher than 0.5, relative to the difference in levels and vice versa. We calculated parameter estimates and fit statistics using the open-source statistical language R following the protocol described by Yuelin [48].

## Results

### Prosocial Behavior in the Costly v s. Cost-free Version

**Figure 2:**
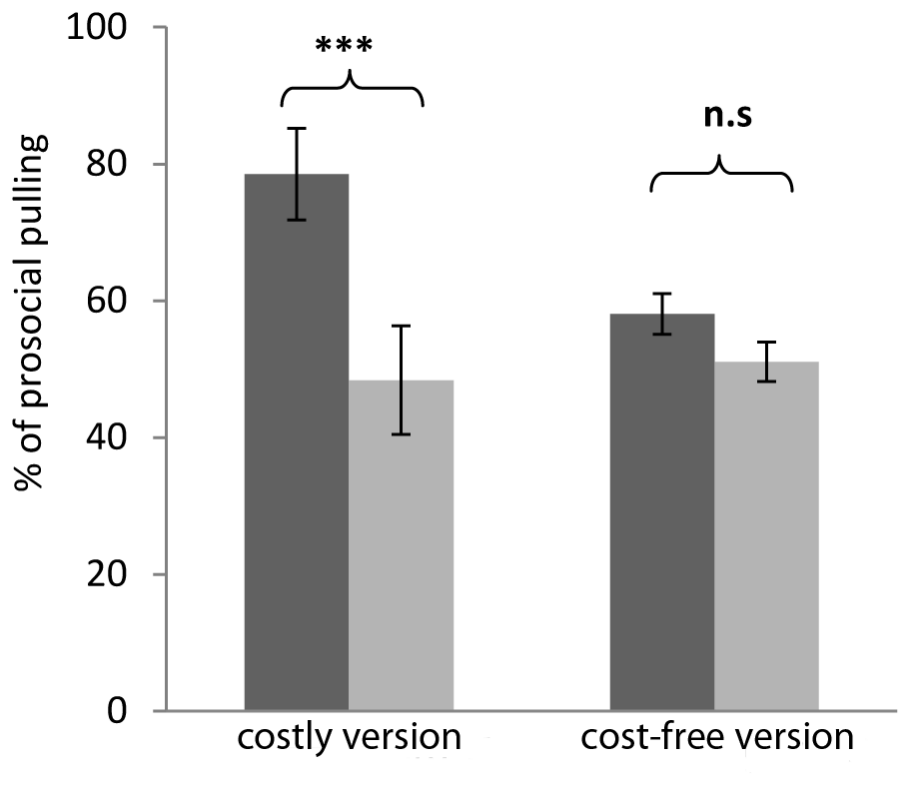
Prosocial effect. Donors’ (n = 31) pulling of the prosocial distribution ([0,1], or [1,1], respectively) in the presence (test condition, dark bar) or absence (control condition, light bar) of a recipient, for both versions of the dictator game. A prosocial effect was present only in the costly version of the game. ***: p<0.001.

In the costly version, the children pulled the prosocial tray more often in the test condition when a partner was present than in the control condition when the recipient compartment was empty, by a margin of 30.1% (SEM = 6.5; one sample t-test on the difference of prosocial pulling in test minus control conditions, t(30) = 4.57 p<0.001). In contrast, no prosocial effect, i.e. no significant difference between test and control condition (mean difference: 6.77%, SEM = 4.78) was found in the cost-free version (t(30) = 1.4, p = 0.167; Figure 2). The prosocial effect was bigger in the costly version (t(30) = 2.75, p = 0.01), and due to more prosocial choices in the costly version in the partner present condition t(30) = 3.07 p = 0.004, whereas prosocial choices in the partner absent condition (control) did not differ between the versions (t(30) = −0.31 p = 0.76).

Prosocial behavior (difference of pulling the prosocial tray in test minus control condition) did not increase with the children’s age in both versions of the game (costly: Spearman’s Rho = 0.088, n = 31, p = 0.97; cost-free: Spearman’s Rho = 0.198, n = 31, p = 0.282). Furthermore, we analyzed the influence of additional factors on prosocial behavior with Generalized Linear Models (GLM), with the presence of a prosocial effect as response variable (i.e. subjects pulling significantly more often in test compared to control condition vs. subjects who did not discriminate), separately for each version of the game. We found no sex differences (costly version: z = −1.30, p = 0.19; cost-free version: z = −0.07, p = 0.94), no effect of whether dyads were composed of same- or different sex partners (costly version: z = 0.13, p = 0.90; cost-free version: z = 0.82, p = 0.41), relationship quality of the dyad as rated by the nursery teachers (costly version: z = 0.93, p = 0.35; cost-free version: z = −0.36, p = 0.72), whether the donor child had siblings (costly version: z = 0.01, p = 1.00; cost-free version: z = 0.01, p = 1.00), older siblings (costly version: z = −0.01, p = 0.99; cost-free version: z = 0.01, p = 1.00) or the sibling position (costly version: z = −0.01, p = 1.00; cost-free version: z = 0.01, p = 1.00). We found no effect of socio-economic status (costly version: z = 0.55, p = 0.59; cost-free version: z = 1.11, p = 0.27) and no order effects, neither for whether the experiment started with test or control session (costly version: z = −1.04, p = 0.30; cost-free version: z = −0.69, p = 0.49) nor for version order (costly version: z = 0.27, p = 0.79; cost-free version: z = −1.31, p = 0.19).

### Attentional, not Motivational Processes Prevent Prosocial Responding in the Cost-free Version

A possible explanation for the contrasting results in the two versions of the game may be that in the cost-free version, the donors simply did not pay attention to the recipient child’s payoff because they were focusing on their own reward. If such attentional limitations are responsible for the lack of prosocial responding in this version, we should see that (i) donors pay less attention to the recipients’ reward dish in the cost-free version compared to the costly version, and thus (ii) pull more impulsively, i.e. with shorter latencies. Furthermore, they should (iii) show less interest in the consequences of their prosocial pulling because this would often result as an unintended, and thus unnoticed, byproduct of getting their own reward.

**Figure 3:**
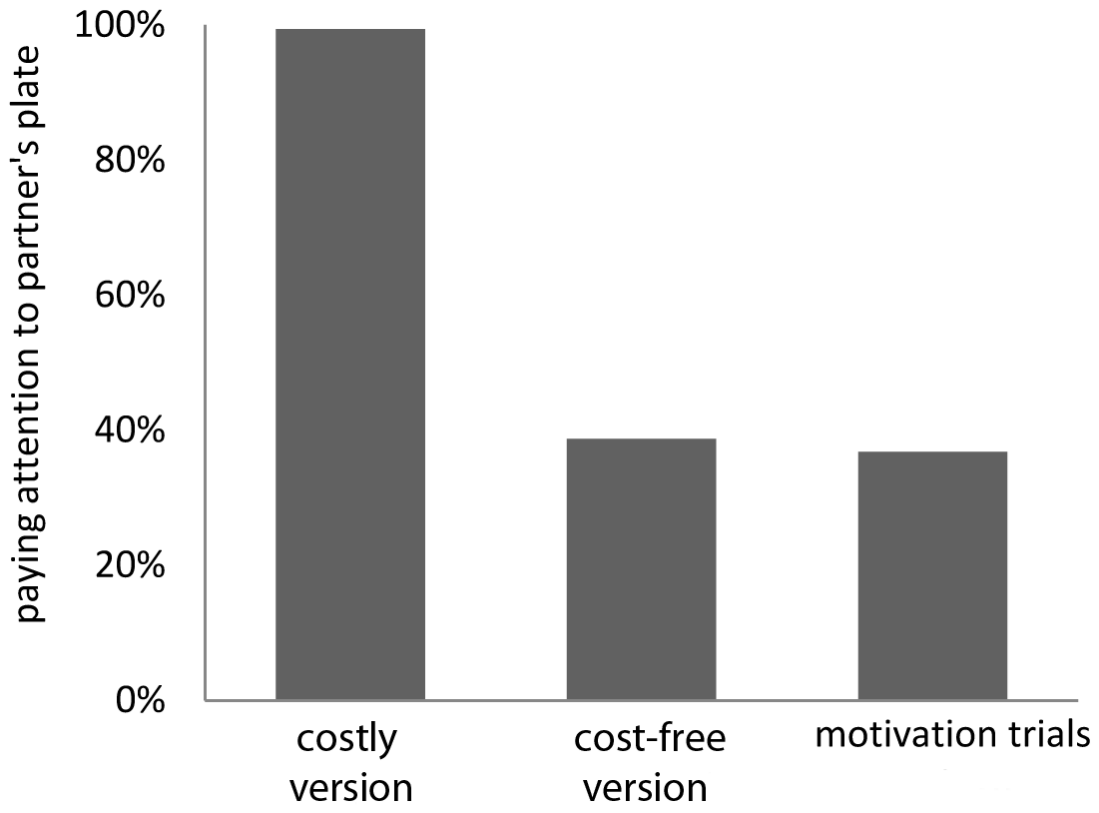
Attention of donors to the partner’s plate before pulling. Percentage of trials when donors looked at the partner’s plate in test sessions of the costly version (reward on partner’s side), the cost-free version (reward on both sides) and during motivation trials (one reward on donor’s side). The presence of a reward for the partner in addition to a reward to the subject herself does not increase attention to the partner’s plate.

First, as shown in Figure 3, donors looked more often at the partner’s plate before pulling when there was no reward on their own side of the apparatus [0,1] than when there was a reward on both sides [1,1], or during motivation trials when there was only a reward on the donor’s side [1,0] (chi^2^ test, χ^2^ = 285.62, df = 2, p<0.001). Indeed, the presence or absence of a reward on the donor’s side was a good predictor of looking at the recipient’s reward position (Generalized Linear Mixed Model, random effect: child, fixed effect: reward, Estimate = −6.127, Std. Error = 0.824, z value = −7.433, p<0.001).

**Figure 4:**
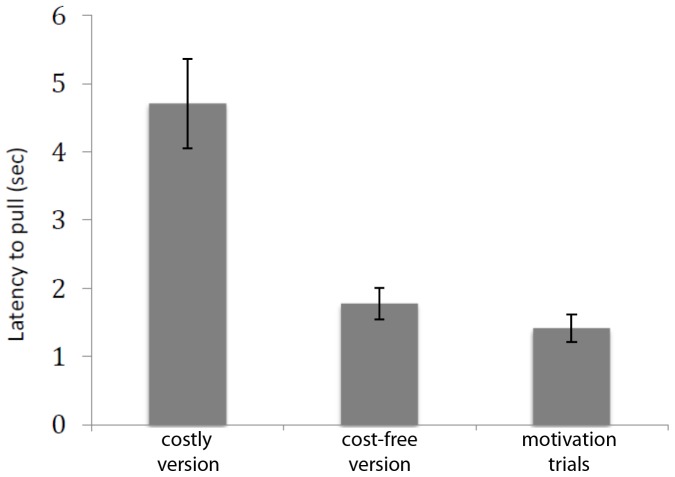
Pulling latencies. Latency to pull the prosocial tray during test trials in the costly version, the cost-free version, and to pull the board baited for oneself during motivation trials. The presence of a reward for the partner in addition to a reward to the subject herself does not increase the latency to pull.

Second, as shown in Figure 4, the donors pulled with longer latencies when only a reward for the recipient was present (test trials of costly version) than when a reward was also present for the subject herself (test trials of cost-free version, permutation test on an one sample t-test statistic, p<0.001, n = 26) or exclusively for the subject herself (motivational trials, permutation test on a one sample t-test statistic, p<0.001, n = 26). Furthermore, the latencies of the test trials of the cost-free version and the motivational trials of the costly version did not differ significantly (permutation test on a one sample t-test statistic, p = 0.184, n = 26). This suggests that whenever the donors could get a reward for themselves, they pulled with short latencies, regardless of what this meant for the potential recipient.

**Figure 5:**
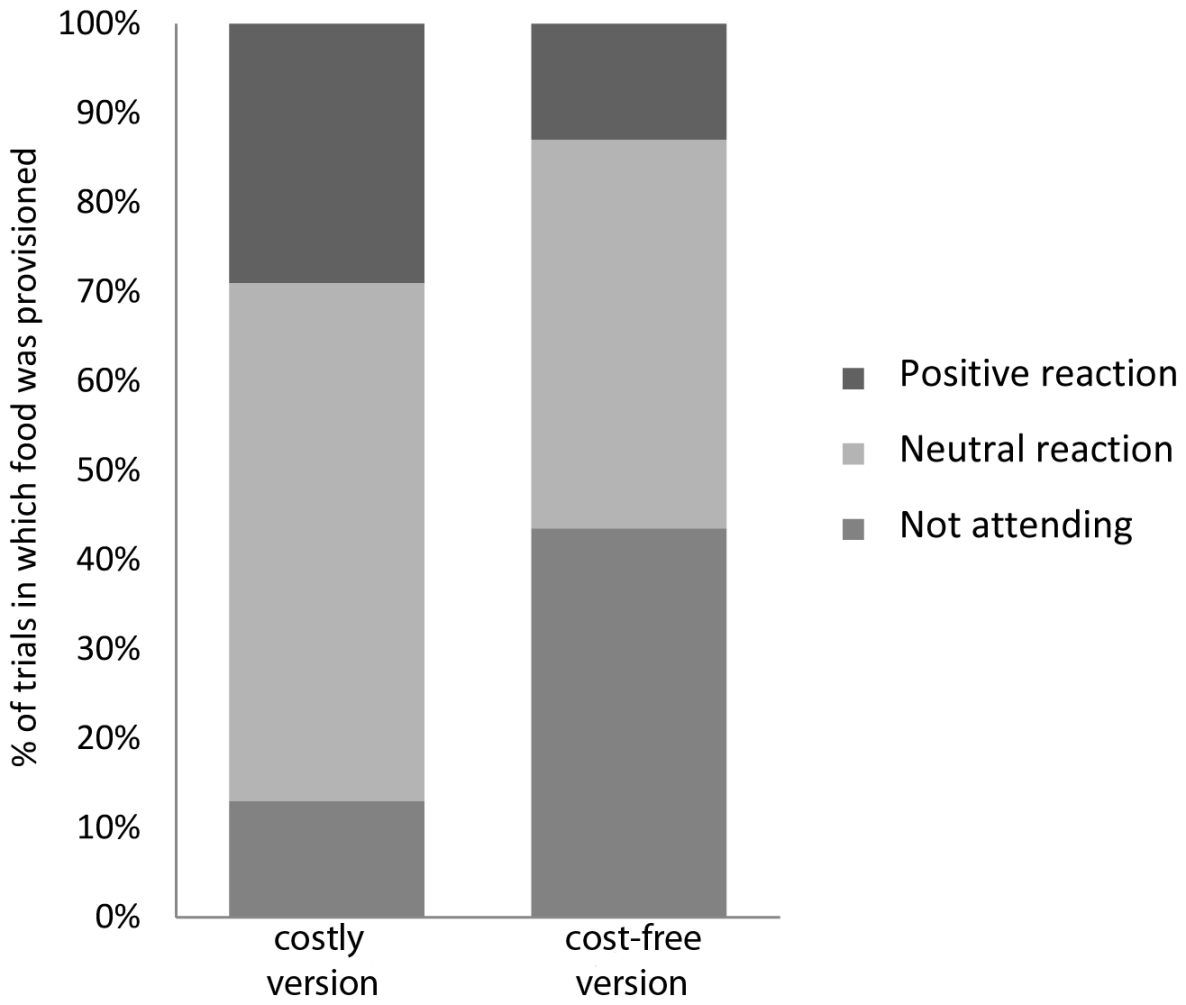
Reactions of the donor children to recipients taking the provisioned reward. The children could either not attend to the recipient at all, attend with a neutral emotional expression, or attend with a positive emotional expression.

Third, as shown in Figure 5, donors also varied in their reactions when the recipient took a reward that had been pulled by the donor for her (146 cases in the costly version, 108 cases in the cost-free version). In the cost-free compared to the costly version, the donors were more likely not to attend at all to the recipient taking the reward (43.5% versus 13% of all cases, respectively), and less likely to attend neutrally without observable emotional reaction (43% versus 58%) and also less likely to attend and show a positive emotional reaction (13% versus 29%; chi^2^ test, χ^2^ = 31.85, df = 2, p-value <0.001).

### Signs of Interest and Requests for Help

**Figure 6:**
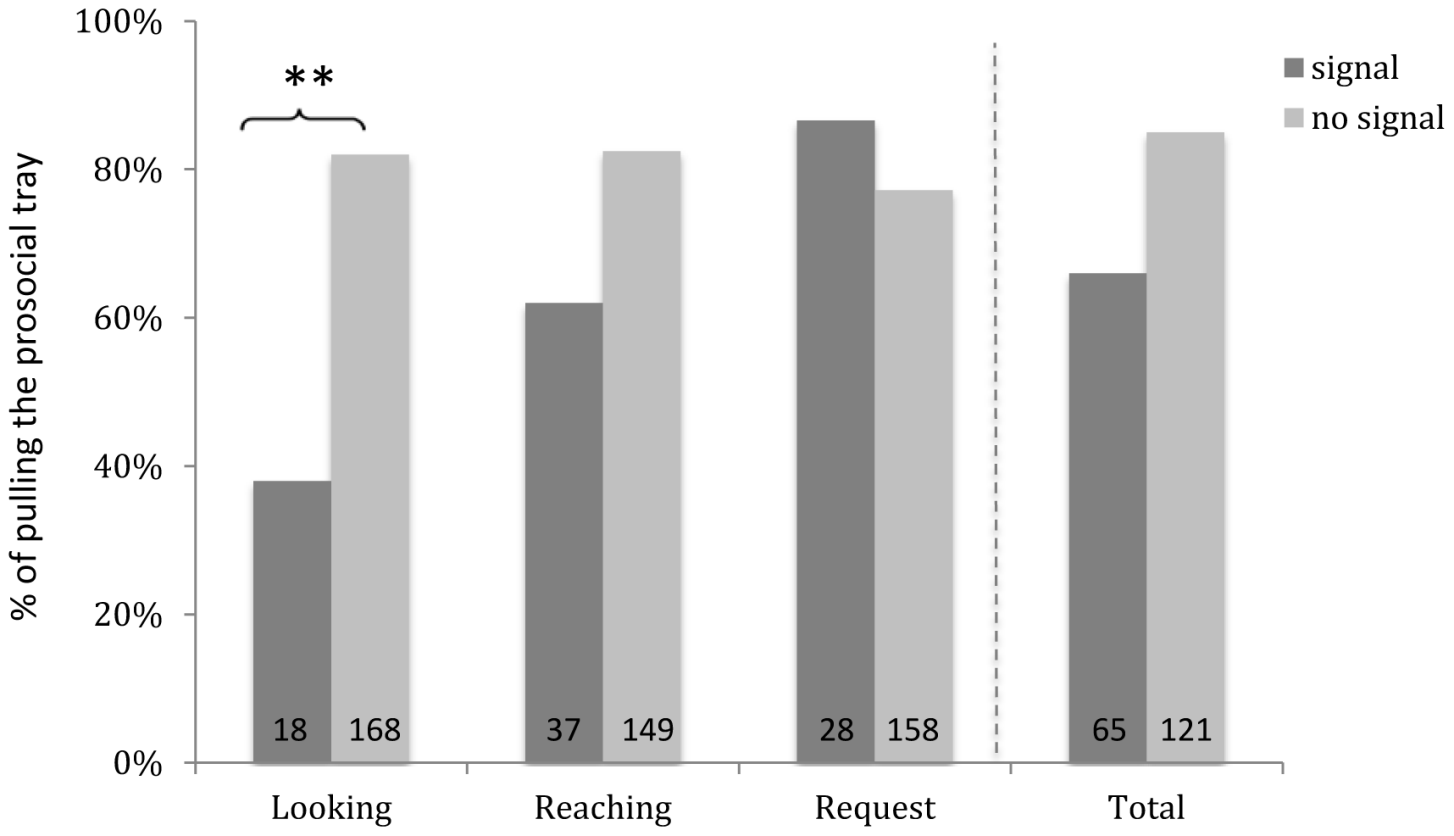
Effect of signs and signals of need on prosocial pulling. Percentage of trials in which prosocial pulling occurred following different kinds of signaling (dark bars) or without signaling (light bars). Figures inside the bars represent numbers of trials. For instance, prosocial pulling occurred in 85% of 121 trials in which no signaling of any kind occurred (total). Looking = recipient looks at reward; reaching = recipient tries to access reward with arm, request = recipient verbally asks for reward.

Signs of interest (looking at and/or reaching for the reward) and requests for help were not necessary to release prosocial choices since in the costly version, 70.5% of all prosocial pulls occurred in the absence of such signs and signals of need. Indeed, both signs of interest (looking at the reward, reaching for the reward) and requests for help did not increase prosocial pulls (Figure 6; Generalized Linear Model on trial level with prosocial pulling as response variables, and looking at reward (*z* = −1.085, *p* = 0.278), reaching for the reward (*z* = −0.888, *p* = 0.375) and request for help (*z* = 0.571, *p* = 0.568) as fixed factors). When we only examine the first test trial per dyad, when any previous influence by recipients can be excluded, 50% of all prosocial pulls occurred in the absence of any sign or signal of need. In these first trials, reaching (*z* = −1.278, p = 0.2) or requests for help 0.791, *p* = 0.43) had no effect on prosocial pulling, but looking at the reward (*z* = −2.139, *p* = 0.032) had a negative effect. In the cost-free version, where we found no overall prosocial effect, the pattern of results was the same.

### Theory of Mind and Prosociality

30 of the 31 donor children attended the four socio-cognitive tests (the youngest donor child [2.14 years] did not want to attend). Each child passed at least one test. The maximum score reached was 4 (cumulative numbers of tests passed). The average child passed 2.17 tests (n = 24). 8 tests had to be excluded for 6 children, because of experimenter’s mistakes or inconclusive answers by the participants.

We validated the ToM measure in two ways. First, we used the Rasch model to validate whether the results from our sample fit a Guttman scale. While the first task, the diverse desire task, was passed by most of the participants (26/30), the number of children who passed the following tasks decreased continuously, with only 2 children (out of 30) passing the final one. The fit statistics (Table 2) indicate that our data correspond to the pattern described in the much larger samples from Wellman and Liu [45] and Kristen et al. [47]. Thus, as in the previous studies, performance in the ToM tasks could be meaningfully ranked according to their difficulty.

**Table 2:** Rasch analyses.

Items	Children who passed (%)	Item difficulty	Standardized infit	Standardized outfit
Content false belief	0.04	3.95	1.26	3.43
Knowledge access	0.42	0.02	1.02	1.18
Diverse belief	0.63	−1.27	0.71	0.56
Diverse desire	0.83	−2.71	1.00	0.78

The higher the “item difficulty” - score, the higher the difficulty level of the item. Fit statistics (standardized infit and outfit values) have an expected value of 0. Values >2.0 indicate a misfit [77].

Second, ToM scores showed a weak sex difference with girls outperforming boys (Mann-Whitney U = 37.5, p = 0.047), as expected. However, due to the narrow range of ages tested, ToM scores were not positively linked with age to a significant degree (all subjects: Rho = 0.335, p = 0.109, n = 24; girls: Rho = 0.311, p = 0.352, n = 11; boys: Rho = 0.251, p = 0.408, n = 13).

Having validated our ToM scores, we asked whether they were correlated with prosocial behavior, i.e. the difference of pulls in test vs control sessions. This was not the case, either in the costly (Rho = −0.028, p = 0.895, n = 24) or the cost-free version (Rho = −0.101, p = 0.64, p = 24). Since mentalizing ability has been hypothesized to impact reactive, rather than proactive prosociality, we also analyzed proactive and reactive prosocial behavior separately. For each donor, we calculated an index of proactive prosociality, i.e. the proportion of all trials without signaling in which donors pulled the prosocial option (where signaled trials were those in which reaching for reward, looking at reward, and requesting help by recipients occurred; signaling was only included if it occurred prior to pulling and was perceived by the donors). Likewise, we calculated the proportion of all trials with signaling in which donors pulled the prosocial distribution. Since part of these pulls may have been motivated by proactive prosociality despite the presence of signaling, we calculated the difference between the two proportions as index for reactive prosociality (i.e. proportion of prosocial pulling in signaled trials minus the proportion of pulling in non-signaled trials). Again, ToM scores were not related to either proactive or reactive prosociality, in the costly (proactive: Rho = -.242, p = 0.254, n = 24; reactive: Rho = 0.021, p = 0.936, n = 18) as well as in the cost-free version (proactive: Rho = 0.305, p = 0.148, n = 24; reactive: Rho = −0.146, p = 0.650, n = 12; the higher sample sizes for proactive pulling are due to the fact that the majority of all food deliveries (70.5%) occurred in the absence of signaling).

## Discussion

We assessed the validity of different versions of prosocial games commonly used with nonhuman primates by presenting them to 2- to 5-year old human children, i.e. at an age when prosocial behavior can be expected. We used an identical experimental setup and procedure as previously used to assess proactive prosociality in marmoset monkeys [11] and implemented two versions of the game, a costly and a cost-free one. Like the monkeys, the children were tested with group members as partners, had to pass pretest criteria to make sure that they had understood the consequences of their choices, and were not verbally instructed to behave prosocially. At the same time, we assessed their level of ToM development with a set of standardized tests [45], [47].

### Some Payoff Distributions Prevent Prosocial Responses Due to Attentional Demands

As expected based on their age (reviewed in [19], [20], [21]), the children behaved prosocially in the costly version of the game, where the donor children could either provide a reward to the partner or not, without ever obtaining anything for themselves. However, prosocial behavior could not be demonstrated statistically with the cost-free payoff-distribution, despite a bigger sample size than in typical nonhuman primate studies that mostly include less than 20 subjects, often less than 10 (for references, see Table 1).

The absence of prosocial behavior in the cost-free version is likely to be a false negative result, because the children did show prosocial behavior in the motivationally even more demanding costly version, where they incur a small cost (pulling the tray), whereas in the cost-free version, they can provide food as a side effect of pulling the tray for themselves. Detailed behavioral analyses suggest that the false negatives occurred because the donor children were oblivious to the donor dish as soon as their own dish was baited: In the cognitively demanding but cost-free version of the game, they were distracted by the opportunity to get their own payoff, and did not pay attention to the recipients’ payoff. Indeed, their overall behavior in the cost-free version of the game was the same as when they could pull food only for themselves (i.e. in motivation trials) with regard to their attentional focus and response latencies. When they nevertheless did provide a reward in the cognitively demanding version, they likely more often did so inadvertently, since they paid less attention to the recipient actually taking the reward, and less often showed a positive emotional reaction to the partner taking the reward compared to the costly version.

The absence of prosocial behavior in the costly version of the game is consistent with a recent result by House et al. [49], who tested 3- to 8-year-old human children in a costly version of a prosocial game previously used with chimpanzees [31]. The children did not choose the prosocial option more often when a partner was present (although a prosocial effect became apparent after statistically controlling for trials that were accompanied by laughter).

Our finding that payoff distributions critically matter whether a prosocial effect can be found is highly relevant, because being able to reliably assess prosociality across species is the crucial precondition for properly identifying the socio-ecological factors that favor the evolution of this trait. The costly payoff-distribution or variations of it based on more or less desirable food, is popular with researchers because it demands only minimal levels of prosociality. Our results, however, suggest that it is exactly this version of the game that this prone to false-negative results, and this may arguably be the case in other species too. Indeed, for some species, negative evidence for prosociality in instrumental pulling taks contrasts with positive evidence from token exchange paradigms [8], [9], and one explanation may be that the latter removes the attentional demands that may prevent prosocial responding in instrumental pulling tasks.

The fact that seemingly trivial differences in experimental design can have far-reaching consequences for our conclusions is not unique to prosociality tasks but has been noticed repeatedly in comparative cognition research in a broad range of domains, as reflected in controversies surrounding visual-perspective taking [50], [51], [52], object-choice tasks (reviewed in [53]) or causality understanding [54], [55], [56], [57]. In each of these cases, similar to the present study, small methodological differences demonstrably lead to fundamentally different conclusions about the presence or absence of an ability; thus, whenever such methodological modifications are confounded with species identity, it becomes impossible to draw conclusions about species differences regarding a specific ability.

Contemporary comparative cognition research thus faces a serious challenge, and the recent recommendation by Silk & House [3] that we urgently need standardized tasks that allow for species comparisons, including humans, not only applies to prosociality tasks. However, the situation may be particularly precarious in tasks that not only involve an experimenter, the subject, and the stimulus material, but in addition also a social conspecific partner, as is the case in prosociality tasks. In such situations, on top of having to manipulate a cognitively demanding apparatus, subjects are involved in two social relationships simultaneously, the one with the experimenter (who tries to be as neutral as possible, but nevertheless will be perceived as a intentional agent, even by small-brained monkeys [58], [59]) and the one with the conspecific social partner. That the simultaneous integration of all these requirements is cognitively demanding is evident from capuchin monkeys, who failed to show targeted helping even though they separately understood the instrumental task and showed a motivation to help in cognitively more simple situations [60].

Ideally, such a standardized approach to assess proactive prosociality relies on very simple, intuitive setup and not only shows whether it is present in a species (for instance, in very specific dyads under highly artificial dyadic experimental situations), but should also provide information about its distribution across age and sex-classes and, most importantly, how prevalent it is under naturalistic situations in the whole-group context (cf. [4]). The recently developed group-service paradigm aims at providing exactly this kind of information, by applying a single experimental setup and procedure to assess proactive prosociality to a wide range of species, testing them in their natural groups and over extended periods of time [61].

### Proactive and Reactive Prosociality

Prosocial responses did not require explicit requests or even the perception of signs or signals of interest in the food by recipients. At least a part of the prosocial responses thus reflect proactive, rather than reactive prosociality [4], as was the case in common marmosets in the same test paradigm.

Signaling of need by recipients decreased, rather than increased, prosocial choices. This is surprising in the context of the child literature, where reactive sharing has been described to be more robust and emerge earlier (e.g. [62]). It is less surprising, however, in the context of the nonhuman primate literature. Signals of interest such as begging and reaching attempts either had no, or even a negative, effect in prosocial games in chimpanzees [8], [63], tamarins [64], and marmosets [11], and arguably only a positive one in capuchin monkeys ([65] decline of prosociality when no visual contact was possible]). This mostly negative influence of signaling need may indicate species differences, but also, and more likely so, that behavioral categories used in these studies lump together functionally highly heterogeneous signals. For instance, unsuccessful reaching attempts may be understood as sign of need and thus elicit prosocial behavior, but may also be perceived as independent solution of the task. Thus, subjects observing the recipient reaching for the food may automatically process this event as “the recipient is accessing the food independently, removing the need for my assistance.” This may also explain the contradictory finding that in another recent study, signaling promoted prosocial responses in human children [62], whereas in the present study, it decreased them. Alternatively, this discrepancy may reflect a more strategic decision to respond to an adult authority figure rather than to a peer (see also below). Disentangling different signs and signals, and whether and how they are perceived by partners, is an important next step for future research and possibly also key to understanding the distribution of prosocial behaviors across nonhuman primates.

Under natural conditions, proactive prosociality of simple acts such as food offering is more prevalent in monkeys, in particular in cooperatively breeding ones with shared infant care, whereas reactive prosociality is more prevalent in great apes, in particular in the form of targeted, instrumental helping (reviewed in [3], [4]). An important factor contributing to this dissociation is likely to be a cognitive one: targeted, instrumental helping arguably both requires an understanding of the partner’s goals and a situational, causal understanding of which behavioral means are most likely to achieve these goals (see also [66], [67]) as well as the ability to integrate such representations in a helping motivation [60]. On the other hand, proactive prosociality, typically measured in food offering contexts may well rely on much simpler cognitive regulation. Under naturalistic conditions, all individuals always need food, and a representation of conspecifics as food-motivated entities may thus be developmentally canalized. Thus, the current pattern suggests that nonhuman primates vary with regard to an intrinsic, proactive helping motivation expressed in cognitively simple contexts, and also that some species such as chimpanzees may be able to behave prosocially in more complex situations due to more powerful cognitive capacities. Notably, these are not necessarily the same species that show particularly high proactive prosociality in the first place, but rather behave prosocially when prompted to do so, by begging or even harassment. At the same time, more general cognitive constraints may prevent other species from showing instrumental helping despite high proactive helping motivation.

### Prosociality and Theory of Mind

Higher levels of explicit ToM understanding did not increase prosocial behavior in the prosocial game, in both versions of the task. Likewise, ToM did not bias children towards more proactive (i.e. the probability to pull prosocially in the absence of signs and signals of need by recipients) or reactive prosocial tendencies (i.e. the probability to pull prosocially as a response to signaling minus proactive prosociality).

One might argue that our sample was too small to conclude that no relationship exists between the level of ToM development and performance in prosocial games. However, the ToM measure employed was valid. In the present study, we were able to replicate the findings by Kristen et al. [47] and Wellman and Liu [45] regarding the increasing difficulty of the tasks. Further consistent with other studies (e.g. [68], [69]), girls slightly outperformed boys. However, we found no significant age effect, presumably due to the narrow age range of the participants which was chosen to minimize the confounding effect of age when investigating the relationship between ToM and prosociality. Because the measure was valid, the absence of any correlation indicates that this relationship, if present, could not have been strong.

Therefore, our results instead indicate that ToM reasoning is not the key factor in eliciting proactive prosociality in young children. This finding is consistent with the result by Sally and Hill [70] who show that prosocial choices in the ultimatum game but not in the dictator game are influenced by false belief understanding. Dictator games are used by economists to assess other-regarding preferences or proactive prosociality, whereas the veto option in ultimatum games adds a strategic dimension [7]. Together, these results are in favor of scenarios that imply a very early, rather indiscriminate onset of (proactive) prosociality during human ontogeny, which is later constrained by strategic decisions based on ToM reasoning (see also [6], [71]). Thus, importantly, ToM reasoning may not only drive decisions towards more prosocial behavior, but also result in decisions when to inhibit a prosocial impulse.

One possible caveat is that this conclusion is based on the use of an explicit ToM measure. ToM development can be traced back to much younger ages [72], and it can therefore be argued that it is such earlier levels that are relevant for the emergence of prosociality. Indeed, mirror self-recognition, for instance, as an early manifestation of self-other differentiation has been linked to the emergence of comforting behavior [22], [23], [73], i.e. one form of reactive prosocial behavior. However, since comforting, helping, and sharing are dissociated developmentally [27], [28] and neurobiologically [29], these must be rather separate phenomena and thus likely be regulated differently. Our experiment arguably assesses sharing behavior (but note that subjects don’t give up any of their own food), and the lack of a relationship is thus not inconsistent with the above mentioned studies.

Whether earlier and more implicit forms of ToM play a role in prosocial game performance cannot be answered based on the present study. However, comparative data suggests it does not, since those early ToM-related abilities evidenced by some species do not predict prosocial game performance across nonhuman primates. Some of these abilities are likely to be present in most nonhuman primates (reviewed in [58], [59], [74]) whereas others, such as mirror self-recognition, can only be found in great apes, but not monkeys [34], including callitrichids [36]. Nevertheless, callitrichids, but not chimpanzees show proactive prosociality in prosocial games. The socio-cognitive abilities that enable mirror self-recognition may thus be relevant for the observed distribution of reactive, instrumental helping across primates, as argued above, but not involved in the regulation of proactive prosociality.

### Conclusion

This study leads to two important conclusions. First, seemingly trivial differences in experimental design of prosociality studies can have far-reaching consequences on our conclusions, including differences regarding payoff distributions as in the present study, but also the nature of the task (instrumental pulling vs. token exchanges), the amount of time provided to respond, or competing demands of attention and affect [75]. Any further advancement in understanding the origin of prosocial behavior critically requires unified approaches that yield comparable data, both at the construct level and at the level of experimental design and procedure.

Second, an emerging pattern of findings in both developmental and comparative psychology is that prosociality is a multidimensional phenomenon. In developmental psychology, different forms prosocial behaviors have been shown to follow separate, non-correlated developmental trajectories [27], [28], to be supported by different neural substrates [29], and to be regulated differently, with ToM, for instance, playing a role in some forms but not in others ([22], [23], [24], [26], [70], [76]; this study). Comparative data now likewise points to the necessity to distinguish different kinds of prosocial behavior, a key distinction being that between proactive and reactive forms of prosociality, as reflected in the role of understanding signs and signals of need for eliciting prosocial behaviors, and the dissociation between performance in prosocial games and targeted helping across species [3], [4]. Merging the findings from the two fields seems an obvious next step, and it is a valid working hypothesis to assume that the distinct forms of human prosocial behaviors may have different phylogenetic histories. Once the evolutionary roots have been identified, we can more easily examine the adaptive function of each kind of prosocial motivation.
